# Construction of a trifunctional cellulase and expression in *Saccharomyces cerevisiae* using a fusion protein

**DOI:** 10.1186/s12896-018-0454-x

**Published:** 2018-07-13

**Authors:** Zi-Lu Liu, Hua-Nan Li, Hui-Ting Song, Wen-Jing Xiao, Wu-Cheng Xia, Xiao-Peng Liu, Zheng-Bing Jiang

**Affiliations:** 1 Hubei Key Laboratory of Industrial Biotechnology College of Life Science, Hubei University, Wuhan, 430062 People’s Republic of China; 2 College of Resources and Environmental Science, Hubei University, Wuhan, 430062 People’s Republic of China; 3 Hubei Collaborative Innovation Center for Green Transformation of Bio-Resources, Hubei University, Wuhan, 430062 People’s Republic of China; 4 Department of Biological Science and Technology, Hubei University for Nationalities, Ensi, 445000 People’s Republic of China

**Keywords:** Lignocellulose, Trifunctional cellulase, Filter paper enzyme activity

## Abstract

**Background:**

Cellulose is the most important component of lignocellulose, and its degradation requires three different types of enzymes to act synergistically. There have been reports of single gene duality, but no gene has been described to have more than two functions. Cloning and expression of fusion cellulases containing more than two kinds of catalytic domains has not been reported thus far.

**Results:**

We synthesized three different cellulase genes and linked the three catalytic domains with a (G_4_S)_3_ flexible linker. The trifunctional cellulase gene (*BCE*) containing three types of cellulase functions was constructed and expressed in *S. cerevisiae* successfully. The β-glucosidase, the exoglucanase and the endoglucanase activity of the trifunctional cellulase BCE reached 16.80 IU/mg, 2.26 IU/mg and 20.67 IU/mg, which was 46.27, 6.73 and 46.20% higher than the activities of the β-glucosidase BG, the endoglucanase CBH and the endoglucanase EG. The filter paper enzyme activity of BCE was higher than those of BG, CBH and EG, reached 2.04 IU/mg.

**Conclusions:**

The trifunctional cellulase BCE was designed based on β-glucosidase BG, endoglucanase EG and exoglucanase CBH, and it possessed β-glucosidase activity, endoglucanase activity and exoglucanase activity simultaneously. The BCE has better filter paper activity, it means the potential practical application.

**Electronic supplementary material:**

The online version of this article (10.1186/s12896-018-0454-x) contains supplementary material, which is available to authorized users.

## Background

Cellulases are mainly composed of various hydrolytic enzymes acting synergistically on cellulosic material which is the main ingredient in lignocellulose [1, 2]. The cellulase market is expected to expand up to $ 400 million per year [3]. With enormous research on these biocatalysts underway, cellulases are extensively used in many industrial fields such as cotton processing [4], paper recycling [5], detergent formulation [6], juice extraction [7], among others [8]. Cellulolytic enzymes are hydrolases that cleave O-glycosidic bonds and are classified according to the sites on the cellulosic substrate upon which they act. Endoglucanases (EG) cleave internal bonds of the cellulosic fiber, exoglucanases (CBH) act on reducing or non-reducing ends, and β-glucosidases (BG) hydrolyze soluble oligosaccharides into glucose [9].

Considering the demand for a synergistic action of multiple enzymes, the expression of a variety of enzymes in a single cell is likely to improve the efficiency of cellulose degradation [10]. There are many ways to express multiple genes in a single cell, but most rely on multiple screening markers and are complicated to operate. Wang et al. constructed a co-expression vector containing two independent expression cassettes based on six restriction enzyme sites and achieved the co-expression of *egv*3 (GenBank: HG313887.1) and *cbh*2 (GenBank: HG313872.1) genes each controlled by a separate promoter in *Trichoderma reesei* [11]. Using a similar method, Gong et al. achieved the co-expression of *eg*3 (GenBank: M19373) and *bgl*1 (GenBank: TRU09580) in *S. cerevisiae* [12]. Liu et al. constructed a bicistronic plasmid (pcbhA-*bgl*) by introducing a 36-nucleotide internal ribosomal binding site ahead of the second gene, *bgl* (Uniprot: O93785), and achieved the co-expression of two kinds of cellulases controlled by a single T7 promoter in *Escherichia coli* [13–15]. Furthermore, co-expressed cellulases and scaffoldin complexes were secreted in *Corynebacterium glutamicum* to enhance saccharification [16]. However, a dockerin domain fusing each cellulase was crucial.

Usually, co-expression can be conducted using either a single plasmid in which it is necessary to select compatible assembly sites to build very large vectors [11], or multiple plasmids, in which case multiple markers and repeated transformations are required [17]. Studies of single proteins containing multiple catalytic domains have attracted great attention in recent years [18, 19]. Mining for this type of enzyme can reduce the complexity of the cloning operation, reduce metabolic stress of the expressing host cell, and enhance the synergistic effect in the process of substrate degradation [20].

Recently, fusion expression strategy, a biotechnology alternative to co-expression, have also been used to express multiple enzymes simultaneously with many advantages including the convenient manipulations of cloning and transforming, the high level of soluble expression, cost-effective purification, and upgraded catalytic capabilities [21]. In addition, it has been confirmed that all individual, fused, and co-expressed endoglucanases and β-glucosidases play a role in hydrolyzing sugarcane bagasse [22].

Previous studies revealed that many natural cellulases have the configuration of modularized domains, such as *Cel9B* (GenBank: AJ133614); from *Paenibacillus barcinonensis* containing an endoglucanase catalytic domain (GH9) and two different cellulose-binding domain (CBDs) [23], and a bifunctional cellulase/xylanase from *Clostridium thermocellum*, called *Ct*CelH (GenBank: ABN52701.1), containing a signal peptide, two catalytic domains, a carbohydrate-binding domain (CBM), and two linkers [20]. Based on this configuration, a versatile protein could be produced by using fusion strategy to connect multiple functional domains. By fusing catalytic domain with the carbohydrate-binding domain (CBM) of another soluble cellulase, the soluble expression of previously insoluble cellulase has been achieved in *E. coli* [24]. However, cloning and expression of fusion cellulases containing more than two kinds of catalytic domains has not been reported thus far [25]. To address this gap, we designed a fusion protein consisting of two linkers and three kinds of cellulase catalytic domains.

*S. cerevisiae* can directly degrade and ferment sugars to ethanol [26]. Heterologous expression of cellulase genes in *S. cerevisiae* has been a focus of investigation to meet the requirement of yeast capable of performing both saccharification and fermentation by expressing cellulases [27]. To reduce the efforts required for cloning and expression, we used a strategy of protein fusion to create a tri-functional enzyme that would enable *S. cerevisiae* to efficiently degrade cellulose. The catalytic properties of the recombinant strains were investigated in order to assess the use of this strategy to produce cellulases in *S. cerevisiae*.

## Results

### Design of the trifunctional single gene

**Fig. 1:**
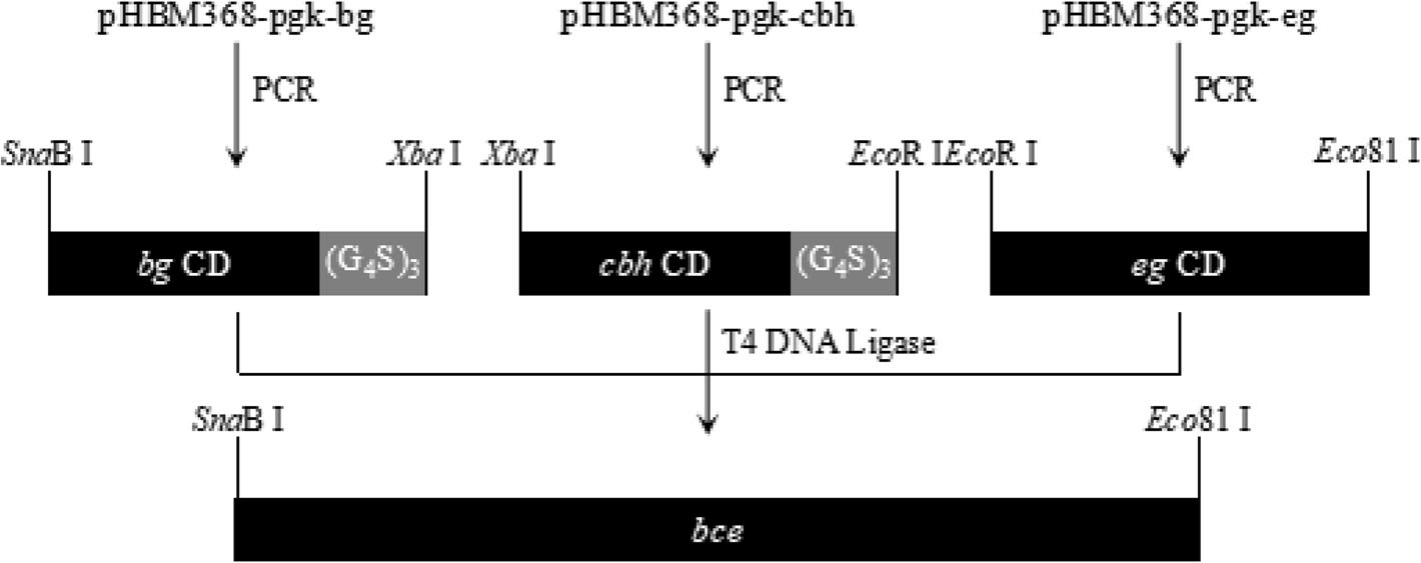
the strategy for construction of the trifunctional single gene.*bg* CD: β-glucosidase catalytic domain, *cbh* CD: exoglucanase catalytic domain, *eg* CD: endoglucanase catalytic domain, (G_4_S)_3_: amino acid linker, *bce*: the trifunctional cellulase gene

In order to construct a single protein with three functional domains of cellulase enzymes, three different cellulase genes without cellulose binding domains were selected from different species. They all had similar optimal reaction temperatures and pH. SignalP 4.1 tool (http://www.cbs.dtu.dk/services/SignalP/) predicted two unambiguous signal peptide cleavage sites before the G_15_ site of the exoglucanase and A_16_ of the endoglucanase. The β-glucosidase has no signal peptide. Inferred from the analysis using PROSITE (https://prosite.expasy.org/) and the Conserved Domain Database (CDD) (https://www.ncbi.nlm.nih.gov/cdd), the different PCR primers were designed for the catalytic domains of the cellulases. At the end of the first two PCR products, the stop codons were removed, while (G_4_S)_3_ linker polypeptides were attached. These primers contained different restriction sites that could splice these three digested PCR products together using T4 DNA ligase generated target fragment showed in Fig. 1.

### Cloning of the trifunctional single gene

**Fig. 2:**
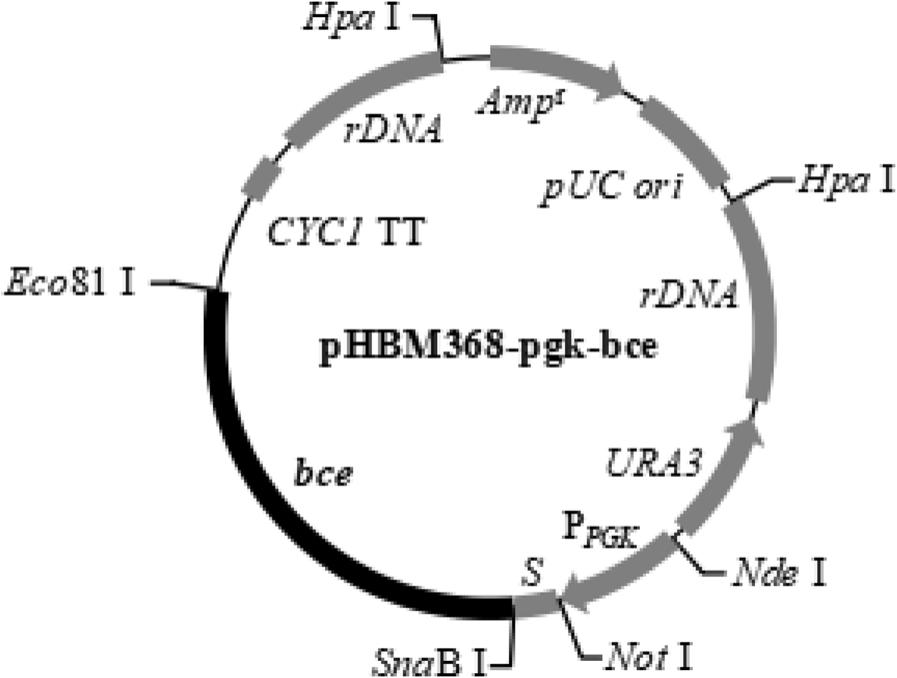
The recombinant plasmid of the expression vector pHBM368-pgk-bce. P_*PGK*_: phosphoglycerate kinase (PGK1) promoter, *S*: α-factor signal sequence, *CYC1 TT*: CYC1 transcription termination signal, *Amp*^r^: ampicillin resistance gene, *Rdna*: ribosomal DNA sequence, *pUC ori*: the replication origin, *URA3*: select maker

The spliced gene, consisting of three catalytic domains, was cloned into the *S. cerevisiae* expression vector pHBM368-pgk. The recombinant plasmid is named pHBM368-pgk-bce (Fig. 2**)**. Transformed strains were initially screened by the restriction enzyme digestion of their plasmids using *Sna*BI and *Eco*81I. The sequencing results of the same plasmid showed it was in line with our design.

### Expression of extracellular recombinant cellulose

**Fig. 3:**
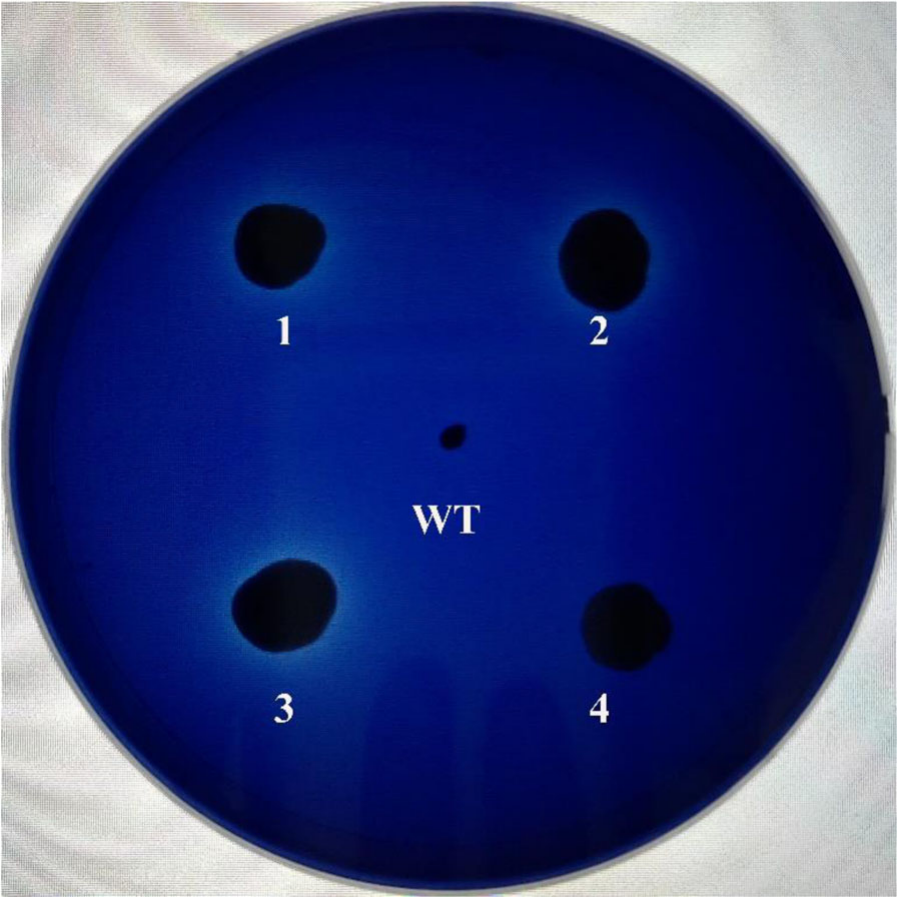
Plate assay of cellulase activity in transformed *S. cerevisiae*. CMC agar plates were stained with trypan blue and enzyme activity was detected by the presence of a halo around the colony. WT: wild-type strain, 1–4: recombinant strains with different cellulase activity

The resulting plasmid, pHBM368-pgk-bce, was linearized with *Hpa* I to promote plasmid integration into the rDNA sequence in *S. cerevisiae* genomic DNA. Transformants were selected based on their ability to grow on minimal agar medium lacking uracil due to the presence of the Orotidine 5′-phosphate decarboxylase gene (*URA3*) in the transforming vector. The integration of the recombinant expression cassette into the yeast genome was confirmed by PCR using the same primers used for gene amplification. Transformants were inoculated onto a chromogenic substrate plate to detect secreted cellulase activity based on the formation of a hydrolysis halo. A clear zone surrounding colonies indicated that recombinant plasmids have been introduced into the host cell (Fig. 3), and that the recombinant enzyme was produced by the expression cassette and secreted into the culture medium. An active strain was chosen and named *S. cerevisiae* BCE. The wild-type strain hardly grown because it could not decompose or utilize cellulose. Finally, to detect the recombinant enzymes (BCE) being produced, culture supernatant collected after a 4-day cultivate were purified as descripted in Methods and analyzed by SDS-PAGE (Additional file 1). The concentration of purified enzyme BCE in the supernatant was 2.23 mg/L, then the purified BCE was used for following assay.

### Recombinant cellulase specificity

**Fig. 4:**
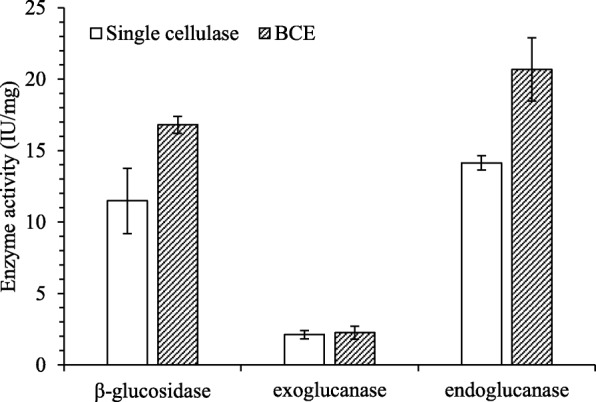
Comparation of enzyme activity based on specific substrate. BCE: trifunctional cellulase BCE, Single cellulase:β-glucosidase BG, exoglucanase CBH and endoglucanase EG. All the hydrolysis experiments were carried out in triplicates

**Fig. 5:**
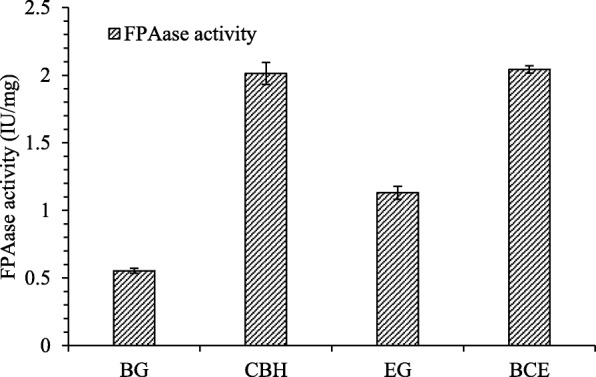
Comparation of FPAase activity between BCE and single cellulase. BG:β-glucosidase BG, EG: endoglucanase EG, BCE: trifunctional cellulase BCE. All the hydrolysis experiments were carried out in triplicates

To understand the substrate specificity of the recombinant enzymes, the activities were assayed using special substrates, namely p-NPG, Avicel and CMC. The β-glucosidase activity of BCE reached 16.80 IU/mg, which was 46.27% higher than BG. The exoglucanase activity of BCE reached 2.26 IU/mg, 6.73% higher than CBH. The endoglucanase activity was 20.67 IU/mg, 46.20% higher than EG. **(**Fig. 4**)**. The results showed that the fusion recombinant cellulase BCE possessed β-glucosidase activity, endoglucanase activity and exoglucanase activity simultaneously. To evaluate the practical application, the Filter Paper Activity (FPase) was assayed. The FPase of BCE reached 2.04 IU/mg, 270.9, 1.5 and 80.5% higher than those of BG (0.55 IU/mg), CBH (2.01 IU/mg) and EG (1.13 IU/mg) **(**Fig. 5**)**. These results indicated that BCE has significant cellulase activity and the potential to degrade complex substrates.

## Discussion

The lignocellulose degradation needs three kinds of cellulases synergistic action. To achieve efficient degradation and biotransformation of lignocellulose, engineering strains with multiple cellulases is a general method. Many works focused on co-expression multiple cellulases [11–13], it’s easy for co-expression two genes in engineering strains. But three kinds of cellulases synergistic action is necessary for efficient degradation of lignocellulose, while co-expression of three genes is difficult. Three or more genes co-expression means low transformation efficient and difficult to achieve recombinants screening. To avoid this problem, constructing a trifunctional cellulase with a single gene could be an effective solution. Many successful studies have been performed on developing the bifunctional cellulase with fusion expression of two different catalytic domains, including endoglucanase catalytic domain fused with β-glucosidase catalytic domain [14, 22], exoglucanase catalytic domain fused with β-glucosidase catalytic domain [15], exoglucanase catalytic domain fused with endoglucanase catalytic domain [25]. However, it has not been reported of an active trifunctional cellulase with three different catalytic domains.

In our studies here, an active trifunctional cellulase BCE was constructed, which presented the activities of β-glucosidase, endoglucanase, and exoglucanase of 16.80 IU / mg, 2.26 IU / mg, and 20.67 IU / mg, respectively, through specific substrate analysis. Compared to the single catalytic domain in the same condition, specific activities of β-glucosidase BG, endoglucanase CBH, and exoglucanase EG were all increased, with the β-glucosidase activity being enhanced the most of 46.27%. These results showed that the active trifunctional cellulase with strengthen catalytic ability could be obtained through the fusion of three different cellulase catalytic domains.

The currently reported bifunctional cellulase with two fused catalytic domains have been expressed mostly in *Escherichia coli* [13–15], *Corynebacterium glutamicum* [16], *Clostridium thermocellum* [18], and *Clostridium cellulovorans* [24]. Comparably, there is no report of using *Saccharomyces cerevisiae* system to express multifunctional cellulase. Although functional studies of these enzymes have all been carried out, it needs to be pointed out that it has no practical significance to compare the different enzymatic properties of these enzymes due to the differences in enzyme activity unit definitions and reaction conditions. In this work, *Saccharomyces cerevisiae* were used as the host cells for expressing trifunctional cellulase BCE, and it was expected that the production of ethanol could be carried out directly by this recombinant *Saccharomyces cerevisiae* strain through using cellulose as a substrate for simultaneous saccharification and fermentation.

The synergistic function of β-glucosidase, endoglucanase, and exoglucanase is necessary for efficient enzymatic hydrolysis of natural cellulose. Filter paper enzyme activity is one of the main parameters to measure the actual application performance of cellulase, which can truly reflect the synergistic effect of enzymatic hydrolysis of the natural cellulose. Through evaluating its enzyme activity against filter paper, the trifunctional cellulase BCE obtained in this study showed a good enzyme activity of 2.04 IU/mg, which is higher than that of a single cellulase expressed in the same system. This result further confirmed that the three different catalytic domains in our constructed trifunctional cellulase BCE could prompt each other, exerting better catalytic function. Comparing with current reports, our work provided an easy approach and methodology for efficient degradation and biotransformation of lignocellulose.

## Conclusion

The trifunctional cellulase BCE was designed based on β-glucosidase BG, endoglucanase EG and exoglucanase CBH, and it possessed β-glucosidase activity, endoglucanase activity and exoglucanase activity simultaneously. The filter paper activity of BCE reached 2.04 IU/mg, a strategy for efficient lignocellulose degradation was provided.

## Methods

### Strains, plasmids, and genes

*Escherichia coli* XL10-Gold was used for cloning and plasmid manipulation. This strain was grown in Luria Bertani (LB) medium (0.5% yeast extract, 1% peptone and 1% NaCl) supplemented with 100 μg/mL ampicillin at 37 °C. The *S. cerevisiae* strain, INVSc1 (His^−^, Leu^−^, Trp^−^, Ura^−^), was used as the host for cellulase production. It was routinely grown in yeast extract peptone dextrose (YPD) medium (1% yeast extract, 2% peptone and 2% glucose) at 30 °C. Synthetic complete minimal medium without uracil (SC-U) medium (1.34% yeast nitrogen base, 2% glucose, 0.01% histidine, 0.01% leucine, 0.01% tryptophan, and 2% agar) was used to screened transformants. An rDNA-mediated integrated expression vector, named pHBM368-pgk, was used for the expression of target genes in *S. cerevisiae* [28]. Three cellulase genes, *eg* (GenBank: CAL48345), *cbh* (GenBank: AGT15838) and *bg* (GenBank: ADY18331), were chemically synthesized (Generay Biotech Co., Ltd., Shanghai), then cloned into pHBM368-pgk, to produce the recombinant plasmids named pHBM368-pgk-eg, pHBM368-pgk-cbh and pHBM368-pgk-bg, respectively.

### Construction of the fusion-expression vector

The strategy used to construct a trifunctional single gene described in this work is depicted in Fig. 1. The DNA fragment encoding the *bg* gene was amplified from pHBM368-pgk-bg by PCR using primers bg-*Sna*BI-F (5′-ATG**TACGTA**AGTAATCCGTTCCCCGAC) and bg-L-*Xba*I-R (5′-A**TCTAGA**CGAGCCACCGCCACCCGACCCACCACCGCCCGAGCCACCGCCACCCCCCAGGCACGCCCCATT) which contain restriction sites (shown in bold) for *Sna*BI and *Xba*I, respectively. The amplicon represents the sequences that encoded the mature BG catalytic domain without the translation start codon or the translation stop codon. A glycine-serine linker, was used as a flexible linker for the construction of fusion protein in the construct: GGGGSGGGGSGGGGS [named (G_4_S)_3_] [29], the reverse coding sequences (underlined in the primer sequence) of which were added in via the bg-L-*Xba*I-R primer. The DNA fragment encoding the *cbh* gene was amplified from pHBM368-pgk-cbh by PCR using primers cbh-*Xba*I-F (5′-ACT**TCTAGA**CAGGGAAATCAGGATTTC) and cbh-L-*Eco*RI-R (5′-A**GAATTC**CGAGCCACCGCCACCCGACCCACCACCGCCCGAGCCACCGCCACCATAAGTGCTATCAATCGGA) which contain restriction sites (shown in bold) for *Xba*I and *Eco*RI, respectively. The amplicon represents the sequences that encode the mature CBH catalytic domain without its native signal peptide, the translation start codon or the translation stop codon. The reverse coding sequences of (G_4_S)_3_ (underlined in the primer sequence) were added in cbh-L-*Eco*RI-R. The DNA fragment encoding the *eg* gene was amplified from pHBM368-pgk-eg by PCR using primers eg-*Eco*RI-F (5′-ATC**GAATTC**CAGTCGCTTTGCGACCAAT) and eg-*Eco*81I-R (5′-ACT**CCTGAGG**CTAGTTGTTTTGTTGGGCGGA) which contain restriction sites (shown in bold) for *Eco*RI and *Eco*81I, respectively. The amplicon represents the sequences that encode the mature EG catalytic domain without its native signal peptide or the translation start codon.

Three amplified cellulase DNA products were ligated together using T4 DNA ligase (TaKaRa, Dalian, China) as per manufacturer’s instructions. The fusion gene was then amplified from the ligation product by PCR using primers bg-*Sna*BI-F and eg-*Eco*81I-R, digested with *Sna*BI and *Eco*81I (TaKaRa, Dalian, China), and then cloned into pHBM368-pgk to generate plasmid pHBM368-pgk-bce. The resulting vector was sequenced by TsingKe Biological Technology Company (Wuhan, China).

### Transformation of *S. cerevisiae*

Three individual-expression vectors and one fusion-expression vector were transformed into *S. cerevisiae* INVSc1 by electroporation after linearization with *Hpa*I. Electrocompetent cells were prepared from a culture growing in log phase, mixed with 10 μg of linearized DNA and electroporated under 1.5 kV, 25 μF, 200 Ω using MicroPulser Electroporator (Bio-Rad, München, Germany). Transformants were selected for their ability to grow on SC-U plates at 30 °C.

### Screening for cellulase activity

Individual transformed colonies were picked from SC-U plates and transferred to YPD plates. Three transformed clones of each individually-expressed cellulase were preliminarily screened for their activity against specific substrates.

Cells expressing the fused cellulase construct were grown at 30 °C for 72 h, then transferred to chromogenic substrate plates containing 0.5% carboxymethyl cellulose (CMC) and 0.02% trypan blue. Cellulase-producing colonies were identified by the presence of a hydrolysis halo around the colony. Individual cellulase-producing colonies were chosen for PCR validation.

### Production and purification of the recombinant enzyme

The colonies with high cellulase activities were further cultured in 50 mL of YPD medium at 30 °C with an agitation rate of 200 rpm for 48 h. Cells were then harvested and resuspended in 50 mL of fresh YPD medium and grown for a further 48 h induction at 30 °C. The culture supernatants were collected by centrifugation at 12,000 rpm for 10 min at 4 °C, followed by ultrafiltration using a Vivaflow® 50 ultrafiltration membrane with a molecular weight cut-off of 30 kDa. Anion exchange chromatography was performed using an ÄKTA Purifier (GE Healthcare, USA). The enzymes were eluted at 0.3 M NaCl. The purified enzymes were analyzed by sodium dodecyl sulfate polyacrylamide gel electrophoresis (SDS-PAGE), diluted with sodium citrate buffer and stored at 4 °C. The concentration of protein was measured using Bradford Protein Assay Kit (Beyotime, Shanghai, China).

### Characterization of enzyme properties

The yeast cells were pelleted by centrifugation at 12,000 rpm for 10 min at 4 °C. The supernatant was used to determine three kinds of cellulase activity. Exoglucanase activity was determined by measuring the glucose yield from 1% (*w*/*v*) Avicel, according to a previously described method [30]. Endoglucanase activity was determined by measuring the glucose yield from a 1% (w/v) CMC solution [31]. β-glucosidase activity was determined by measuring the concentration of p-nitrophenol (pNP) liberated from p-nitrophenyl β-d-glucopyranoside (pNPG) [32]. Colorimetric detection was performed with SpectraMax M5 Microplate Reader (Molecular Device, USA) at 540 nm and 405 nm for glucanase and β-glucosidase, respectively. One unit (IU) of enzyme activity was defined as the amount of enzyme required to liberate 1 μmol of glucose or p-nitrophenol per minute.

Filter Paper Activity (FPase) Assay was used to examine the ability of the cellulases to degrade complex cellulosic structures in line to the modified standard procedure [33]. Each of the reaction systems contained two pieces of Whatman No. 1 filter paper (approximately 5 mg/mL), which had been soaked in citrate-phosphate buffer for one day. One unit (IU) of FPase was defined as the amount of enzyme required to liberate 1 μmol of glucose per minute.

All the hydrolysis experiments were carried out in triplicates. The error bars were calculated and displayed in results.

## Additional file

Additional file 1: **Figure S1.** SDS-PAGE analysis of the recombinant multifunctional cellulase. (JPG 453 kb)

## Abbreviations

BCE: Trifunctional cellulase gene (BCE)
CBD: Cellulose-binding domain
CBM: Carbohydrate-binding domain
CMC: Carboxymethyl cellulose
FPase: Filter paper enzyme
PCR: Polymerase chain reaction
