# Supplementary figures and images for: Construction of a trifunctional cellulase and expression in Saccharomyces cerevisiae using a fusion protein
Source: BMC Biotechnol. 2018 Jul 13;18:43. doi: 10.1186/s12896-018-0454-x (PMC6044064; doi:10.1186/s12896-018-0454-x)

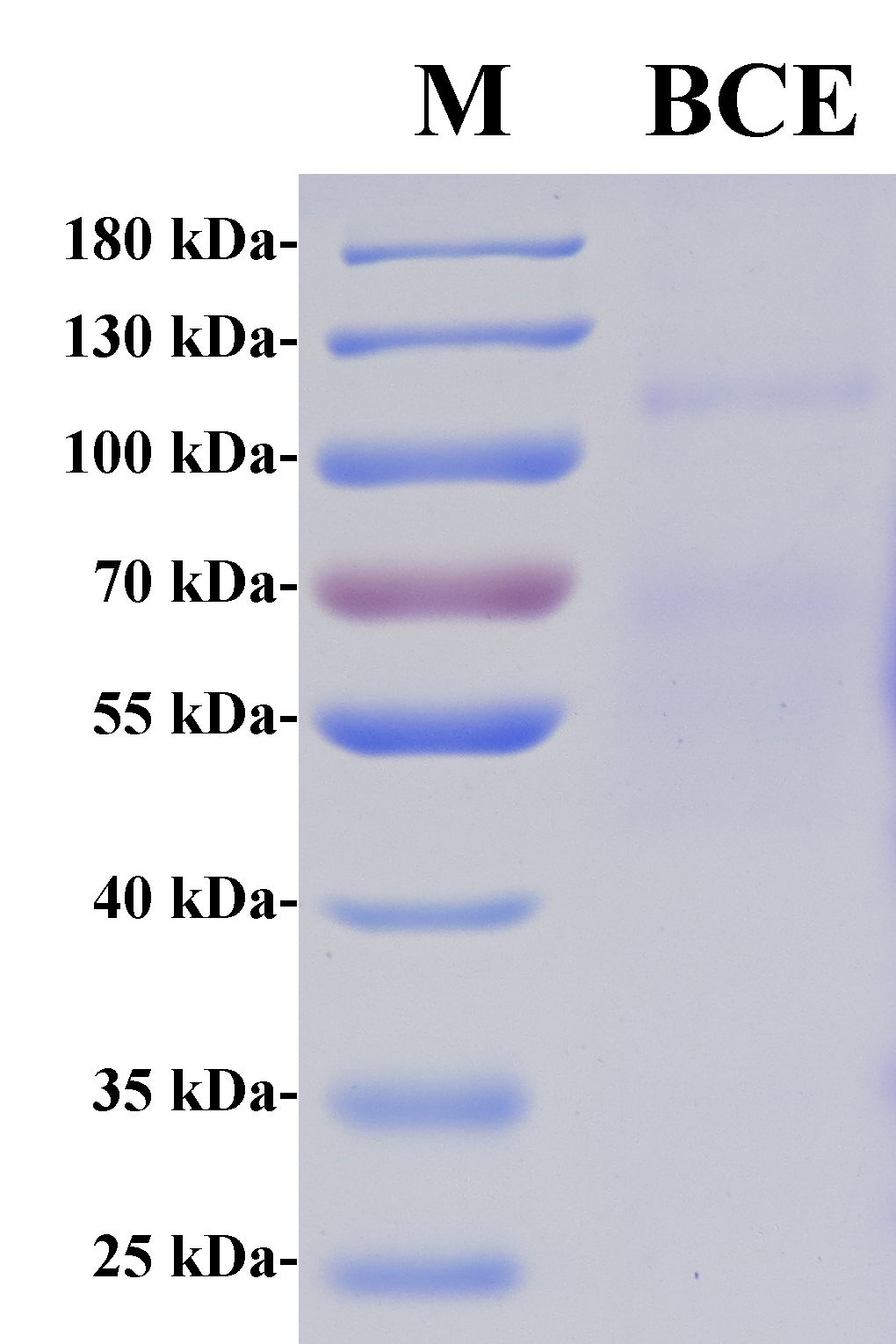

Supplement: Supplementary file 1 — Figure S1. SDS-PAGE analysis of the recombinant multifunctional cellulase. (JPG 453 kb) [file 12896_2018_454_MOESM1_ESM.jpg]
